# Acute G6PD deficiency haemolytic crises with associated methaemoglobinaemia in a patient with typhoid

**DOI:** 10.4102/sajid.v41i1.771

**Published:** 2026-01-13

**Authors:** Jake P. Jacob, Midhun T. John, Gilad Mensky, Lior Chernick

**Affiliations:** 1 Department of Infectious Diseases, Faculty of Health Sciences, Helen Joseph Hospital, Johannesburg, South Africa; 2 Department of Infectious Diseases, Faculty of Health Sciences, University of the Witwatersrand, Johannesburg, South Africa

**Keywords:** G6PD deficiency, haemolytic crises, methaemoglobinaemia, typhoid

## Abstract

Glucose-6-phosphate dehydrogenase (G6PD) deficiency is an X-linked disorder that increases red blood cell susceptibility to oxidative damage. While haemolysis is common, methaemoglobinaemia is a rare complication. A 39-year-old man developed both conditions, triggered by *Salmonella typhi* infection. Diagnosis was supported by Heinz bodies and blister cells on blood smear. Methylene blue and high-dose ascorbic acid were relatively contraindicated because of ongoing haemolysis and renal dysfunction.

**Contribution:**

This case highlights the importance of considering methaemoglobinaemia in G6PD deficiency during infections and adapting treatment when standard therapies are unsuitable.

## Case presentation

The patient was a 39-year-old Mozambican citizen who had been residing in Johannesburg. He reported multiple visits to Mozambique bi-annually, usually 3–4 weeks at a time. He had no known comorbidities and was previously well. He reported multiple previous episodes of malarial infection, the last of which was 2 years prior, treated with oral medication.

He had been in Mozambique for the last 3 weeks, where he developed fever and vomiting, which lasted 10 days, followed by non-bloody diarrhoea. He presented at a local clinic in Mozambique, where he was diagnosed with malaria and received outpatient treatment. He received four tablets twice a day for 3 days – which is in keeping with our presumption of artemether and lumefantrine (‘Coartem’). He returned to Johannesburg during his last day of treatment. On previous occasions, he would improve within days; however, after the completion of the treatment, he experienced worsening dyspnoea, fever, jaundice and macroscopic haematuria for 5 days. Thereafter, he presented to Helen Joseph Hospital Casualty.

On examination, the patient was pale, the blood pressure was 168/98 mmHg, the pulse rate was 121 beats per minute, the temperature was 37.8 °C and the respiratory rate was 36 breaths per minute using accessory muscles, indicating significant respiratory distress. This is in association with an oxygen saturation of 85% on ambient air, which did not improve on face mask oxygen at a fraction of inspired oxygen (FiO_2_) of 60%. On systemic examination, the liver was enlarged (2 cm), with a smooth surface, and was non-pulsatile. On inspection of the urine sample, there was macroscopic haematuria confirmed as blood on the urine dipstick.

Laboratory investigations showed haemolysis evidenced by a haemoglobin decrease from 10 g/dL on admission to 4 g/dL (after 3 days), with a macrocytosis of 104 fL, aspartate aminotransferase (AST) of 2425 U/L (normal range 15–40), an unconjugated hyperbilirubinaemia (total bilirubin 35 µmol/L and conjugated bilirubin 17 µmol/L) and a lactate dehydrogenase (LDH) of > 3500 U/L (normal range 100–190) (see Table 1).

**TABLE 1:** Laboratory investigations on admission.

Parameters	Values
White cell count (× 10^9^/L)	6.98
Red cell count (× 10^9^/L)	3.15
Haemoglobin (g/dL)	10.9
Haematocrit (L/L)	0.329
Mean cell volume (fL)	104.5
Platelet count (× 10^9^/L)	120
C-reactive protein (mg/L)	215
International normalised ratio	1.30
Activated partial thromboplastin time (s)	27.1
Potassium (mmol/L)	4.6
Sodium (mmol/L)	137
Chloride (mmol/L)	100
Bicarbonate (mmol/L)	18
Urea (mmol/L)	34.5
Creatinine (μmol/L)	856
eGFR (mL/min/1.73 m^2^)	6
Total bilirubin (μmol/L)	35
Conjugated bilirubin (μmol/L)	17
Total protein (g/L)	67
Albumin (g/L)	36
Alkaline phosphatase (U/L)	145
Gamma-glutamyl transferase (U/L)	96
Alanine transaminase (U/L)	439
Aspartate transaminase (U/L)	2425
Lactate dehydrogenase (U/L)	> 3500

eGFR, estimated glomerular filtration rate.

**FIGURE 1:**
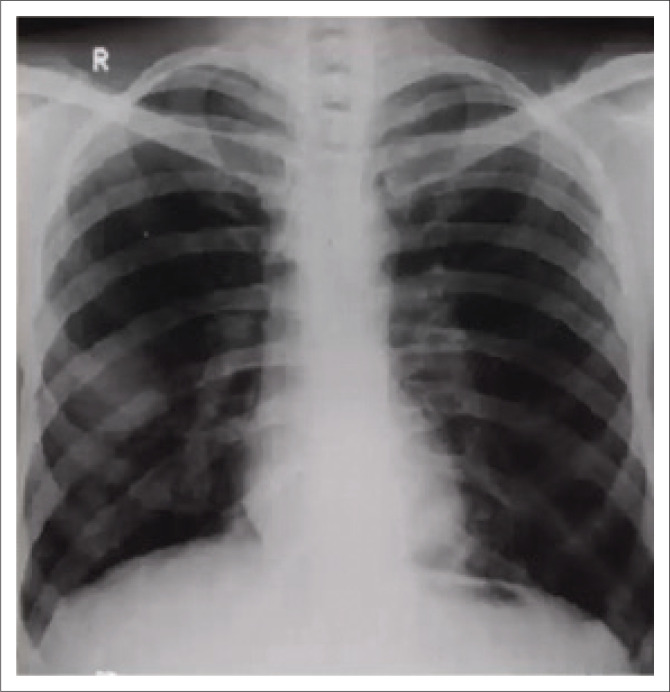
Chest radiograph.

During the first 3 days of admission, the patient developed temperature spikes between 38.4 °C and 38.8 °C. The patient was in respiratory distress with a respiratory rate of 24–36 breaths per minute. There were no obvious respiratory signs on clinical examination or abnormalities on the chest X-ray, as seen in Figure 1. The patient was admitted to the high care unit and was initiated on intermittent haemodialysis.

The first arterial blood gas determination demonstrated a discrepancy between the reading of the pulse oximeter (85%) and the blood gas co-oximeter, which showed an oxygen saturation of 99.6%. The difference in the saturations can be explained by the methaemoglobin level of 12.4% demonstrating that this patient has methaemoglobinaemia, as seen in Table 2.

**TABLE 2:** Arterial blood gas measurements on arrival of the patient to the hospital.

Parameters	Values
pH	7.449
pCO_2_ (mmHg)	35.7
pO_2_ (mmHg)	213
Lactate (mmol/L)	0.9
Haemoglobin (g/dL)	4.0
Methaemoglobin (%)	12.4
Base excess (mmol/L)	0.8
Saturated O_2_ (%)	99.6†

Note: Pulse oximeter 85% on face mask oxygen supplementation (fraction of inspired oxygen = 60%).

pH, potential of hydrogen; pCO_2_, partial pressure of carbon dioxide; pO_2_, partial pressure of oxygen.

† , arterial on O_2_.

**FIGURE 2:**
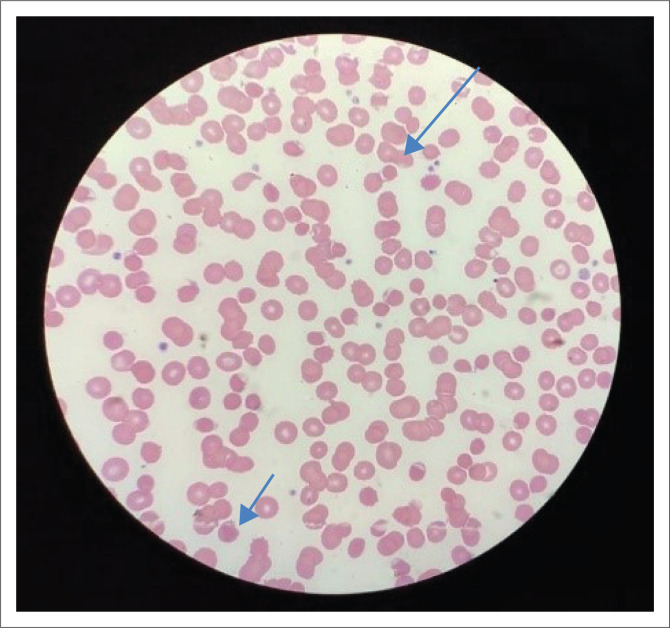
Blood smear demonstrating blister cells (indicated by blue arrows).

**FIGURE 3:**
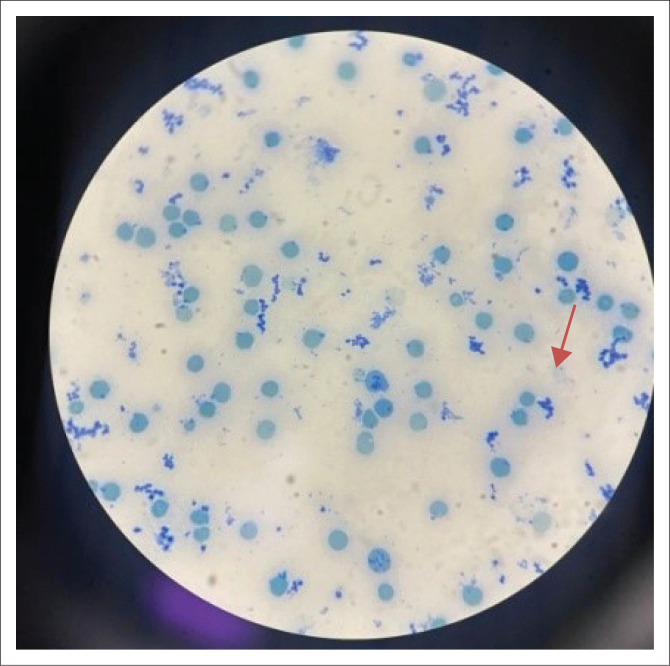
Blood smear demonstrating Heinz bodies (indicated by red arrow).

On day 2 of hospitalisation, an urgent blood smear was done, showing significant blister cells and Heinz bodies. In association with the haemolysis, glucose-6-phosphate dehydrogenase (G6PD) deficiency was suspected (see Figure 2 and Figure 3). The smear was negative for malaria and confirmed on a pan-malarial polymerase chain reaction.

**FIGURE 4:**
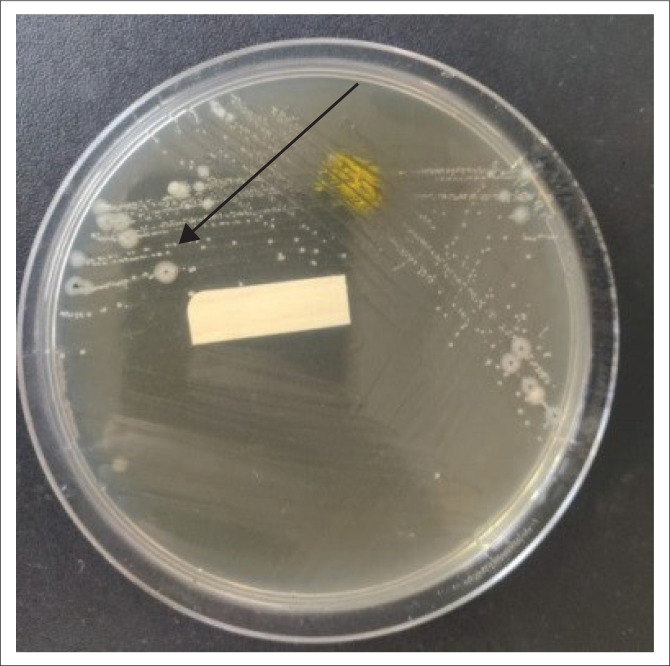
On day 6 of admission, colourless and transparent colonies with black centres (black arrow) shows positive for *Salmonella typhi* on *Salmonella Shigella* agar as a culture medium.

**FIGURE 5:**
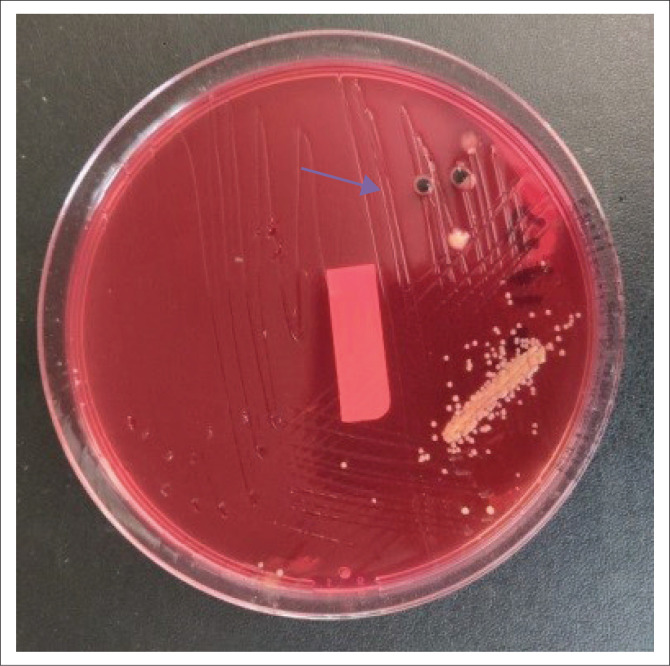
On day 6 of admission red colonies with black centres (blue arrow) shows positive for *Salmonella typhi* on xylose lysine deoxycholate agar as a culture medium.

**FIGURE 6:**
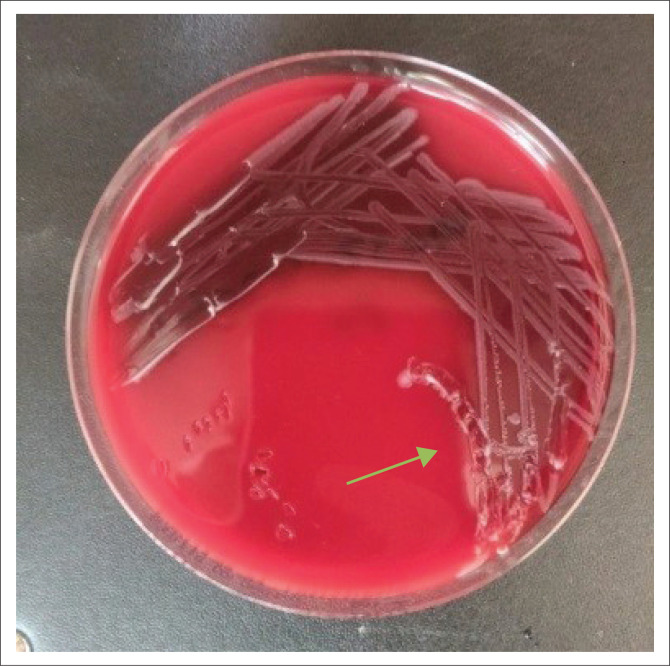
On day 6 of admission grey colonies (green arrow) shows positive for *Salmonella typhi* on blood agar as a culture medium.

A septic screen was done as part of the initial workup, including blood, urine and stool cultures. On day 6 of admission, the stool culture was positive for *Salmonella typhi*, as seen in Figure 4 (colonies are colourless and transparent with black centres as seen by black arrow), Figure 5 (colonies are red with black centres as seen by blue arrow) and Figure 6 (colonies are grey as seen by green arrow).

Supportive management, including intermittent haemodialysis, supplementary oxygen, fluids, blood products and targeted antibiotic (ceftriaxone), was the mainstay of treatment. Specific treatments for methaemoglobinaemia, such as methylene blue and high-dose ascorbic acid, were both relatively contraindicated because of the ongoing haemolysis and renal dysfunction in this clinical case. The patient subsequently improved and was discharged with full recovery. The patient was counselled on precautionary measures for G6PD deficiency, such as avoiding fava beans, mothballs and the use of aspirin. He was also counselled to let healthcare workers know if seeking medical advice that he has a G6PD deficiency.

## Discussion

The patient is a Mozambican citizen with a recent travel history to Mozambique. The prevalence of G6PD deficiency is greater in this region (up to 16%)^1^ and is associated with protection against malarial infection. Typhoidal *Salmonella* is also endemic in this region. This patient has a G6PD deficiency and presented with an acute haemolytic episode secondary to acquiring typhoidal *Salmonella* while in Mozambique. The G6PD deficiency further caused the methaemoglobinaemia resulting in his respiratory distress, although the sepsis in itself, renal failure and anaemia were contributing factors. The renal dysfunction was caused by many factors stemming from the intravascular haemolysis, sepsis and methaemoglobinaemia. Other possible precipitants of the acute haemolysis from the G6PD deficiency could have been toxin or drug induced. However, the patient denied drug or toxin use besides the presumed artemether and lumefantrine (‘Coartem’) given by his local clinic.

### Glucose-6-phosphate dehydrogenase deficiency and methaemoglobinaemia

Methaemoglobinaemia is a condition in which there is diminished oxygen-carrying capacity of circulating haemoglobin because of the conversion of Fe^2+^ to the oxidised ferric state Fe^3+^.^2^ In the ferric state, haemoglobin is unable to bind to oxygen. The oxygen dissociation curve is also shifted to the left, which increases the affinity of the haemoglobin for oxygen. Oxygen release to tissues is impaired. The clinical presentation of methaemoglobinaemia is varied, the most common features being cyanosis, dyspnoea and hypoxaemia refractory to supplementary oxygen. The arterial blood gas co-oximeter will show a normal oxygen saturation, whereas the pulse oximeter oxygen saturation is falsely low as a result of the pulse oximeter not being able to accurately differentiate between oxyhaemoglobin and methaemoglobin. Pulse oximeters detect oxygen levels in the blood by shining two different wavelengths of light (red and infrared) through the finger and measuring how much light is absorbed. In G6PD deficiency, there is a portion of haemoglobin that is oxidised to methaemoglobin, which absorbs light at similar wavelengths to oxyhaemoglobin and deoxyhaemoglobin, which causes the pulse oximeter to underestimate the oxygen saturation.^2^ Other clinical sequelae include fatigue, seizures, drowsiness, arrhythmias and death. The level of clinical severity is influenced by several factors: percentage of methaemoglobin, rate of production of methaemoglobin, underlying anaemia and cardiac and respiratory reserve of the patient. The methaemoglobin level was 12.4% with a haemoglobin (Hb) level of 4.0 g/dL, which gives 0.496 g/dL of methaemoglobin (Table 2). This level is generally normal in a healthy individual, but given the severe anaemia, this would indicate severe methaemoglobinaemia leaving the patient with functional haemoglobin of 3.5 g/dL. The saturation gap is the difference between the pulse oximeter oxygen (SpO_2_) and the arterial saturation on the blood gas machine (SaO_2_). A saturation gap above 5% suggests the presence of a haemoglobinopathy such as methaemoglobinaemia, sulfhaemoglobinaemia and carboxyhaemoglobinaemia. In this patient, the saturation difference was 14.6%.

Methylene blue is an antidote used in methaemoglobinaemia. Treatment is not contraindicated but remains controversial in patients with G6PD deficiency, where the NADPH-MetHb reductase reduces methylene blue to leukomethylene blue by using the nicotinamide adenine dinucleotide phosphate (NADPH) from the G6PD-dependent hexose monophosphate shunt. This can precipitate a G6PD deficiency haemolytic crisis. However, it is noteworthy to mention that the dose of methylene blue associated with precipitating a G6PD deficiency haemolytic crisis is over 5 mg/kg, which is more than twice the recommended dose.^2^ Our patient was already haemolysing in association with typhoid, and hence, methylene blue was not utilised. Additional treatment options include high-dose ascorbic acid (up to 10 g/dose), exchange transfusion and hyperbaric oxygen therapy.^2^ High-dose ascorbic acid with underlying renal dysfunction can predispose to hyperoxaluria by enhancing urinary excretion of oxalate and resulting in stone formation.^2^

### Glucose-6-phosphate dehydrogenase deficiency and typhoid

There is an interesting dual association between G6PD deficiency and typhoid, where typhoid is a known precipitant of haemolytic anaemia in G6PD-deficiency patients, and G6PD-deficient patients have an increased susceptibility to typhoid fever.^3^ Crowell et al. found a threefold increase of typhoid in G6PD-deficient patients compared to a control group. Typhoid fever has been shown to cause a transient depression of erythrocyte G6PD activity. A study in 1982 in South East Asia found that G6PD levels during the third week of typhoid fever were approximately 30% lower than after recovery.^4^ Hepatitis is also highly associated with haemolysis in G6PD-deficient patients, and this may be the case for typhoid as well. The exact mechanism of typhoid-induced G6PD deficiency haemolysis is unclear. It is known that oxidative compounds exposed to red blood cells increase the pentose pathway activity. In G6PD-deficient patients, there is a lower threshold to deal with these toxic metabolites resulting in both the methaemoglobinaemia and haemolysis. Khajehdehi et al. showed that fresh plasma in the acute stage of typhoid fever diminished glucose utilisation in both normal and G6PD-deficient erythrocytes.^5^ This supports the assumption that typhoid infection is able to alter G6PD enzyme properties reducing the pentose phosphate pathway activity and further reducing glucose utilisation. There are multiple mechanisms postulated as to how typhoid achieves this, including direct effect of the microorganism, accumulation of oxidative compounds and erythrocyte damage by activated complement. During infection, there is increased production of hydrogen peroxide generated by phagocytosing leukocytes, which possibly supersedes the threshold of conversion by the already deficient G6PD enzyme reducing its ability to convert this acid into water.^4^ It has been reported that erythrocytes carry receptors for activated complement. Therefore, complement-activated immune complexes (typhoid microbes and antibodies) attract granulocytes, further increasing oxidative damage to the red blood cell.

## Conclusion

Our patient had a higher risk compared to the general South African population of having a G6PD deficiency as he is native to Mozambique. His recent travel to Mozambique increased his risk of contracting typhoid because of its endemic nature in the area. Furthermore, the G6PD deficiency increased his risk of acquiring typhoid, and the typhoid increased his risk of having a haemolytic crisis and subsequent methaemoglobinaemia.

Glucose-6-phosphate dehydrogenase deficiency is rare in South Africa. This case report highlights the importance of always being vigilant for G6PD deficiency and ensuring that one looks at the specific presentation as displayed above.
